# The mean point of vergence is biased under projection

**DOI:** 10.16910/jemr.12.4.2

**Published:** 2019-09-09

**Authors:** Xi Wang, Kenneth Holmqvist, Marc Alexa

**Affiliations:** 1 TU Berlin, Germany; 2 Regensburg University, Germany; 3 Nikolaus-Kopernikus University, Poland

**Keywords:** eye movement, eye tracking, fixations, gaze, vergence, bias, calibration

## Abstract

The point of interest in three-dimensional space in eye tracking is often computed based on intersecting the lines of sight with geometry, or finding the point closest to the two lines of sight. We first start by theoretical analysis with synthetic simulations. We show that the mean point of vergence is generally biased for centrally symmetric errors and that the bias depends on the horizontal vs. vertical noise distribution of the tracked eye positions. Our analysis continues with an evaluation on real experimental data. The estimated mean vergence points seem to contain different errors among individuals but they generally show the same bias towards the observer. And it tends to be larger with an increased viewing distance. We also provided a recipe to minimize the bias, which applies to general computations of gaze estimation under projection. These findings not only have implications for choosing the calibration method in eye tracking experiments and interpreting the observed eye movements data; but also suggest to us that we shall consider the mathematical models of calibration as part of the experiment.

## Introduction

Humans tend to direct both of their eyes at roughly the same point in 3D space. Binocular saccades and smooth pursuit between objects in a 3D scene often exhibit vergence, which means that two eyes move in opposite directions [1] for fixation to coincide with the intended object. In other words, vergence is the movement of both eyes towards or away from each other, depending on the relative change from the previous to the current target. It is often assumed that the fixation points of the two eyes are perfectly aligned but it has been shown that the eyes first diverge before they converge at the gaze point during fixations [2, 3]. Studies on binocular coordination of eye movements during reading show that fixation points of two eyes vary during reading and disparity in both horizontal and vertical directions were observed [4, 5]. There is considerable variation among participants ability to fixate the same point in depth, depending on their eye dominance and squinting, or even strabismus (when the weak eye is off-target). In addition, measurements of pupil positions with video-based eye-trackers are very sensitive to variations in pupil dilation [6], which leads to uncertainty over measurements of vergence.

Many approaches have been proposed to estimate the direction of gaze for each eye in physical space, based on recorded pupil positions by eye-tracking devices. Using these gaze vectors, it is possible to reconstruct the gaze point on real three-dimensional stimuli by intersecting one or both rays with the fixated object in space, assuming its geometry is known [7, 8, 9]. Alternatively, we can attempt to find the point where the two vectors intersect with each other in space [10, 11, 12, 13, 14], but in 3D space two gaze vectors typically do not intersect.

However, even if the observer experiences looking at a point in space with both eyes, the eye rays provided by the eye-tracker contain error, for a variety of reasons:

- data from eye-trackers have an inaccuracy (systematic error) and introduce imprecision (variable error, or noise) onto the signal [15, 16, 17].
- the inaccuracy is not constant, but varies with pupil size [18] and quantization of the CR in the eye camera [19].
- ideally, human gaze direction is controlled to bring the object into the fovea centrals [20], which has a non-negligible extent of 1.5–2°.
- it is well-known that in binocular vision, many observers have a dominant eye which is more accurately directed towards the target (in about 70 % of the cases, the right eye) and a weaker eye, which may be considerably off-target [2, 3, 21], which is called binocular disparity (strabismus in extreme cases).
- the resulting unknown and likely non-linear function mapping from tracked pupil and CR centers in the eye video to lines of sight is approximated using low order polynomials [15, 22, 23].

For these reasons, the two projected eye rays produced by eye-trackers are generally skewed and have no common intersection point in space. In order to calculate a point that approximates the expected intersection of the rays, the most natural and commonly employed solution is to compute the point that has the smallest distance to both rays in 3D. Here we call this point the vergence point. We derive the necessary equations for this computation next and then use it for simulating the reconstruction of vergence points in the presence of systematic (accuracy) and variable (precision) errors. We then develop the mathematical description of inaccuracy and imprecision of gaze vectors, that are used to simulate the effect on estimated intersection point of inaccuracy (offsets) and imprecision (noise). [6] present human data of vergence error as an effect of inaccuracy from changes in pupil dilation. In this work, we focus on the effect of imprecision and present recorded human vergence data that validate the simulation on noise. Finally, we present a method to better estimate the intersection of eye rays and reconstruct the position of the fixated object in 3D, given noisy vergence data. Note that the whole analysis applies to eye tracking not only in space but also on flat surface, as long as the underling projective mapping is used in the model.

## Part 1: Mathematical model and simulation

When recording binocular gaze in 3D, the two gaze vectors can be thought of as originating in the centers of the two eyes of the observer. Two vectors in three-dimensional space are generally skew, i.e. they have no common (intersection) point. For the reasons mentioned in the introduction, the two gaze vectors are commonly far from intersecting. In order to assign a point of interest given two gaze vectors that have no intersection point, the dominant strategy is to compute the point in 3D space that has the smallest sum of squared distances to the two gaze lines. Here we show how to compute distances of a point to a line by using the formulation of projector, and then how to find the point of interest.

**Figure 1:**
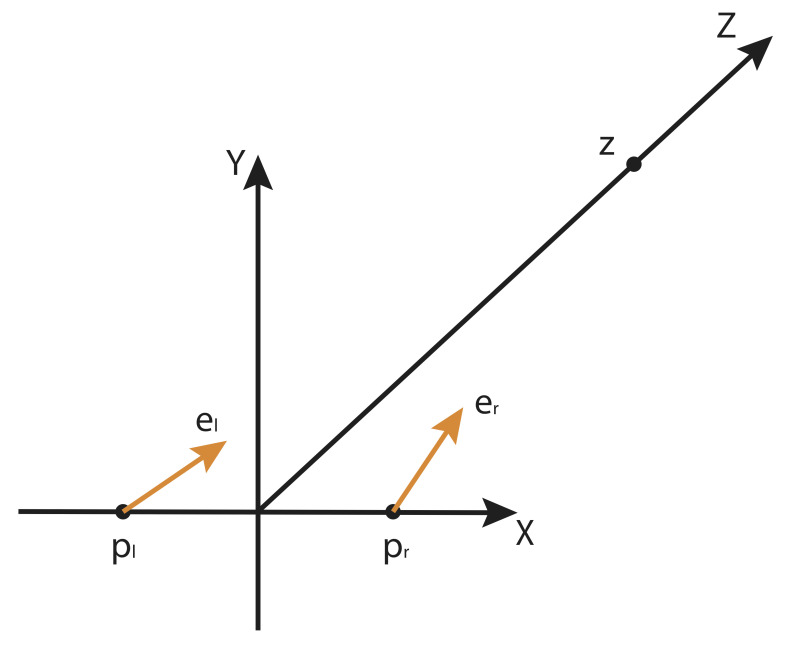
Geometric setup. **p**_l_ and **p**_r_ are the centers of the left and right eyes, and **e**_l_ and **e**_r_ are gaze vectors of unit length, i.e. the eye rays. We want to calculate the point **z** with the minimal distance to both rays.

Choose the coordinate system such that centers of the eyes are displaced symmetrically from the origin along the first coordinate direction. The up direction defines the second coordinate direction and target is placed on the third direction pointing away from the observer. This means the centers of the left and right eyes are

(1) $$\left(\begin{array}{c} -a\\ 0\\ 0 \end{array}\right)=p_{l},\;\left(\begin{array}{c} a\\ 0\\ 0 \end{array}\right)=p_{r}.$$

Here, and in the following, boldface lower-case characters denote column vectors in Euclidean space. Define an up direction as ***u*** = (0,1,0)*^T^*. We refer to the first coordinate axis as horizontal and the second one (i.e. along the up direction) as vertical. Objects of interest located at ***z*** ∈ ℝ^3^ are displaced from the eyes mostly along the third coordinate axis, i.e. ***z*** = (≈ 0, ≈ 0, *z*). Then the normalized (unit-length) vectors from eye to interest point are

(2) $$e_{l}=\frac{z-p_{l}}{\left\|z-p_{l}\right\|},\;e_{r}=\frac{z-p_{r}}{\left\|z-p_{r}\right\|},$$

where the subscripts *l* and *r* refer to the left and right eye. Normalization ensures that the vectors have unit length:

(3) $$1=\left\|e_{l,r}\right\|={\left\|e_{l,r}\right\|}^{2}=\langle e_{l,r},e_{l,r}\rangle =e_{l,r}^{T}e_{l,r}.$$

Note that latter notation for the inner product follows from **e**_l/r_ being a column vector and the usual conventions for matrix multiplication; we use this notation in what follows.

Given only the positions of the eyes and unit gaze vectors, we want to compute the point closest to both eye rays. One way to do so is measuring the squared distances to the rays and finding the point that minimizes them. Let’s first consider a ray through the origin. We define it by specifying a unit direction vector ***v*** ∈ ℝ^3^, ***v*** = ***v****^T^****v*** = 1. Then the points on the ray in the direction ***v*** are given by *λ****v***, where *λ* ∈ ℝ is a scalar parameterizing the ray.

Consider the (symmetric) matrix ***V*** = ***I*** − ***v****^T^****v***. Here ***I*** denotes the 3 × 3 identity matrix (we generally use uppercase bold-face letter for matrices) and ***v****^T^****v*** is an outer product, following directly from the common rules for matrix multiplication:

**Table:** 

Notation	Meaning
**p**_l,r_ ∈ ℝ^3^	the position of left/right eye
a ∈ ℝ	half of the distance between two eyes
***u*** ∈ ℝ^3^	the up direction vector
***z*** ∈ ℝ^3^	object of interest
*z* ∈ ℝ	third element of the object of interest ***z***
**e**_l,r_ ∈ ℝ^3^	normalized eye ray directions
***E***_l,r_ ∈ ℝ^3×3^	projectors of the gaze vectors
d ∈ ℝ	Euclidean distance between two points
**ε**_l,r_ ∈ ℝ^3^	variable error vector of each eye
$e_{\text{l},\text{r}}^{\prime }\in {\mathbb{R}}^{3}$	noisy eye ray directions
*η_l_*_,_*_r_* ∈ ℝ	horizontal noise
*ν_l_*_,_*_r_* ∈ ℝ	vertical noise
*p*	Gaussian distribution
Σ_l,r_ ∈ ℝ^3×3^	Covariance matrix
*σ* ∈ ℝ	standard deviation

(4) $$V=I-\left(\begin{array}{c} v_{0}\\ v_{1}\\ v_{2} \end{array}\right)\left(\begin{array}{ccc} v_{0} & v_{1} & v_{2} \end{array}\right)=\left(\begin{array}{ccc} 1-v_{0}^{2} & -v_{0}v_{1} & -v_{0}v_{2}\\ -v_{0}v_{1} & 1-v_{1}^{2} & -v_{1}v_{2}\\ -v_{0}v_{2} & -v_{1}v_{2} & 1-v_{2}^{2} \end{array}\right).$$

Multiplication of this matrix with any point *λ****v*** on the ray yields

(5) $$V\lambda v=\left(I-vv^{T}\right)\lambda v=\lambda \left(v-vv^{T}v\right)=\lambda \left(v-v\right)=0,$$

while multiplying with a vector ***w*** ∈ ℝ^3^ of arbitrary length but orthogonal to ***v***, i.e. ***v****^T^****w*** = 0, yields

(6) $$Vw=\left(I-vv^{T}\right)w=w-vv^{T}w=w.$$

So the matrix ***V*** annihilates components in direction ***v*** and leaves directions orthogonal to ***v*** unchanged. It is commonly called a *projector* for the direction ***v***. Similarly define the projectors for the gaze vectors ***E****_l_*, ***E****_r_*.

**Figure 2:**
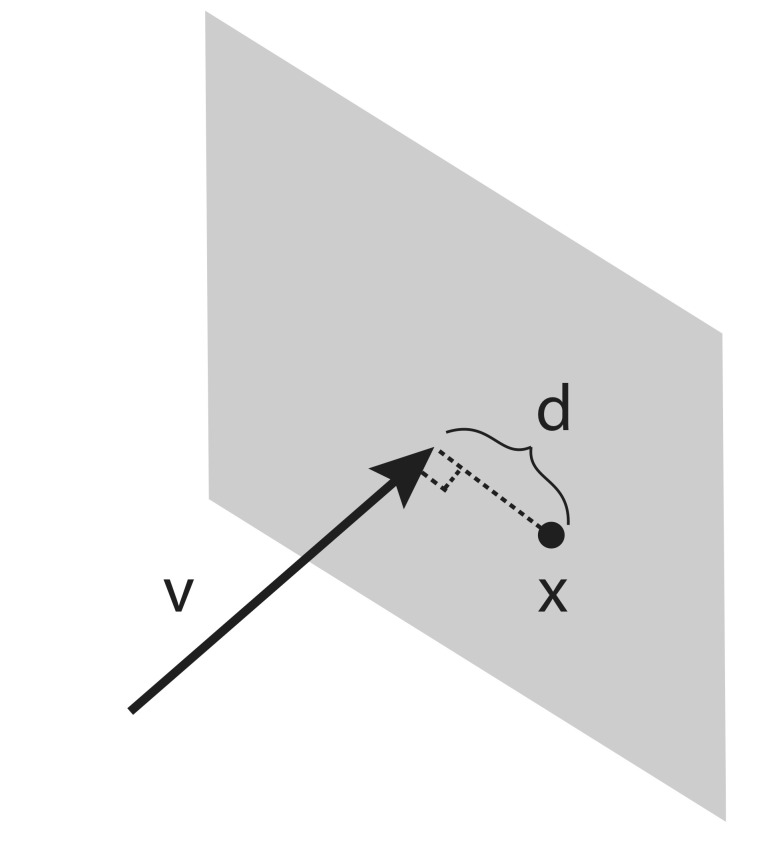
Scalar variable d represents the distance from point **x** to vector **V** that lies in the plane perpendicular to **V**.

Multiplying a point ***x*** ∈ ℝ^3^ from the left and the right, i.e. ***x****^T^****Vx***, results in taking the inner product of the component orthogonal to the vector or, in other words, measuring the squared distance of ***x*** to the line along ***v*** through the origin. If the line is not through the origin, all we need to know is a point p on the line. Then we translate everything so that ***p*** is in the origin, meaning we get the squared distance of ***x*** to the line along ***v*** through ***p*** as

(7) $${\left(x-p\right)}^{T}V\left(x-p\right).$$

With this way of measuring the distance to a ray, the sum of the squared distances to the eye rays for any point ***x*** in space can be written as:

(8) $$d^{2}\left(x\right)={\left(x-p_{l}\right)}^{T}E_{l}\left(x-p_{l}\right)+{\left(x-p_{r}\right)}^{T}E_{r}\left(x-p_{r}\right).$$

To find the point in space that minimizes this sum of squared distances compute the gradient of this function (with respect to ***x***)

(9) $$\nabla d^{2}=2E_{l}\left(x-p_{l}\right)+2E_{r}\left(x-p_{r}\right),$$

and set it to zero:

(10) $$\left(E_{l}+E_{r}\right)x=E_{l}p_{l}+E_{r}p_{r}=\left(E_{r}-E_{l}\right)p_{r}.$$

In this way the point of interest ***x*** is defined as the solution of a 3 × 3 linear system. The system has a unique solution as long as the sum ***E****_l_* + ***E****_r_* is non-singular. Each of the two matrices ***E****_l_*_,_*_r_* has a one-dimensional kernel: the ray direction ***e****_l_*_,_*_r_* is an eigenvector with zero eigenvalue. If the two gaze vectors are parallel, then the projectors are identical and ***E****_l_* + ***E****_r_* = 2***E****_l_* = 2***E****_r_* is singular. This is quite intuitive, as there is no unique point with smallest distance to two parallel lines.

If the eye rays are not parallel, however, the sum ***E****_l_* + ***E****_r_* is non-singular. This is also geometrically intuitive as there is a unique point minimizing the squared distances to the two lines; and this fact can be proven rigorously ([24] Corollary 2.5). Thus, the point of interest is defined as

(11) $$e_{l}\neq \lambda e_{l},\;\lambda \in \mathbb{R}\Rightarrow x={\left(E_{l}+E_{r}\right)}^{-1}\left(E_{r}-E_{l}\right)p_{r}.$$

### Eye ray errors

Firstly, we introduce variable error (imprecision, noise) **ε**_l_, **ε**_r_ into the eye rays, with separate horizontal (*η*) and vertical (*ν*) noise as well as separate noise for left and right eyes:

(12) $$\varepsilon_{l}=\left(\begin{array}{c} \eta_{l}\\ \nu_{l}\\ 0 \end{array}\right),\;\varepsilon_{r}=\left(\begin{array}{c} \eta_{r}\\ \nu_{r}\\ 0 \end{array}\right).$$

While errors are usually represented in terms of angular deviation (i.e. radians), for small enough values the linear approximation *sin ϕ* ≈ *ϕ* is very good and adding the error vectors to the eye ray vectors has the same effect as rotating the eye rays. Including renormalization this yields:

(13) $${e^{\prime }}_{l}=\frac{e_{l}+\varepsilon_{l}}{\left\|e_{l}+\varepsilon_{l}\right\|},\;{e^{\prime }}_{r}=\frac{e_{r}+\varepsilon_{r}}{\left\|e_{r}+\varepsilon_{r}\right\|}.$$

With this model we simulate how noise affects the computation of the vergence point. Following the results in [25] (which show that noise in eye trackers are mostly Gaussian distributed), we use a zero-mean Gaussian distribution, i.e.

(14) $$p(\varepsilon_{l,r})={\left|2\pi \Sigma_{l,r}\right|}_{+}^{-1/2}\exp(-\frac{1}{2}\varepsilon_{l,r}^{T}\Sigma_{l,r}^{-1}\varepsilon_{l,r}).$$

with the horizontal and vertical deviations being uncorrelated (meaning noise distribution in each direction varies independently)

(15) $$\Sigma_{l,r}=\left(\begin{array}{ccc} \sigma_{\eta_{l,r}}^{2} & 0 & 0\\ 0 & \sigma_{\nu_{l,r}}^{2} & 0\\ 0 & 0 & 0 \end{array}\right).$$

In this setup, error vectors can be generated by simply drawing the components *η*_*l*,*r*_, *ν*_*l*,*r*_ independently from univariate normal (Gaussian) distributions with zero mean and standard deviation $\sigma_{\eta_{l,r}}\;,\sigma_{\nu_{l,r}}$. univariate normal (Gaussian) distributions with zero mean and standard deviation $\sigma_{\eta_{l,r}}\;,\sigma_{\nu_{l,r}}$.

**Figure 3:**
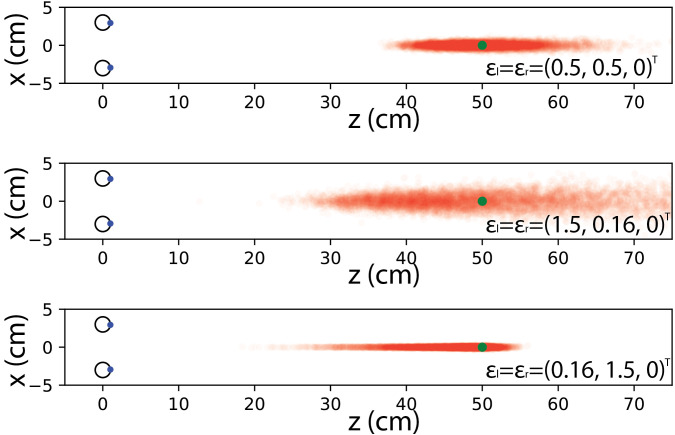
Vergence points from eye rays distorted with zero-mean normally distributed errors (i.e. noise). Distance between two eyes is 6 cm and the object is at the distance of 50cm. Top row: the noise distribution has standard deviation of 0.5 degrees in both horizontal and vertical direction. Despite the relatively small error, the variation in depth is quite large. Middle row: standard deviation in vertical direction is only 0.16, but in horizontal direction it is 1.5. The noise in horizontal direction gets larger and the mean estimated point of vergence is shifted towards larger depths. Bottom row: standard deviation is 1.5 degrees in vertical direction and 0.16 in horizontal direction. Horizontal noise is small, but the mean estimated point of vergence shifts significantly towards the viewer. Same number of samples is drawn in each simulation. 10,000 points are current shown in the figure.

A point of interest ***z*** defines the unbiased eye rays ***e***_*l*,*r*_, which align with the lines of sight. To generate points of vergence in the presence of precision error, we draw error vectors *ε*_*l*,*r*_ (i.e. the added noise), modify the eye rays accordingly and reconstruct the vergence point using the linear system above. The python script given in the appendix does exactly this. It outputs the mean vergence and offers graphical output, such as the one shown in Figure 3. We chose three standard deviations of 0.16°, 0.5° and 1.5° for the Gaussian distributions of noise (which are motivated by experimental data described later). 0.5° is the commonly used threshold of the average calibration accuracy of each marker, 0.16° corresponds to the average precision of fixations and 1.5° corresponds to difficult eye tracking situations for example when observers wear glasses. The simulation shows a large error in direction of depth – this is quite intuitive given the short base line relative to the distance to the object.

More severely, the mean vergence point appears to be biased systematically towards or away from the observer. This is clearly visible when the noise distribution has different variance in horizontal and vertical directions as shown in Figure 3: if variance is larger in the horizontal direction, the mean vergence points shifts away from the viewer; if variance is larger in the vertical direction, the mean vergence point shifts towards the viewer. Table 1 shows detailed simulation results when the distance between observer and the fixation target is considered as another variable. We perform the simulation when the fixation target is placed at three different distances, namely 50 cm, 70 cm and 110 cm. The further away the target is, the larger the bias of the mean vergence point contains. Note that this bias is non-linear due to the underlining projective relation. Within each condition, the bias is mainly in depth but large variation in horizontal direction leads to large bias while the same amount of variation in vertical direction corresponds to smaller bias.

**Table 1:** Simulation errors with target placed at three different distance 50 cm, 70 cm and 110 cm. σx and σy represents the standard deviation in horizontal and vertical directions measured in degrees. ${\bar{\text{p}}}_{\text{z}}$ is the mean position of estimated vergence points in depth. Averaged distance errors and standard deviations are reported in cm with x represents the horizontal direction, y the vertical direction, and z in depth. Notice that the small baseline of distance between the eyes leads to large bias when the target point is further away. On average, the errors of the estimations are 27% (50 cm), 35% (70 cm), and 49% (110 cm) in percentage of the corresponding distances.

Target distance (cm)	*σ_x_* (deg)	*σ_y_* (deg)	${\bar{p}}_{z}$ (cm)	$\bar{error\;}$ (cm)	$\bar{std}$ (cm)	${\bar{error}}_{x}$ (cm)	${\bar{error}}_{y}$ (cm)	${\bar{error}}_{z}$ (cm)	${\bar{std}}_{x}$ (cm)	${\bar{std}}_{y}$ (cm)	${\bar{std}}_{z}$ (cm)
50	1.5	1.5	38.6	14.0	8.1	0.6	0.6	13.9	0.5	0.5	8.2
50	1.5	0.16	41.3	12.8	8.6	0.6	0.6	12.8	0.5	0.6	8.7
50	0.16	1.5	36.8	13.2	2.5	0.06	0.5	13.2	0.04	0.4	2.6
70	1.5	1.5	49.5	25.4	17.1	0.7	0.7	25.4	0.7	0.7	17.1
70	1.5	0.16	56.1	23.9	28.6	0.8	0.09	23.9	0.9	0.1	28.7
70	0.16	1.5	45.8	24.2	4.7	0.1	0.7	24.2	0.05	0.5	4.7
110	1.5	1.5	63.5	54.7	44.8	1.0	1.0	54.6	1.1	1.1	44.8
110	1.5	0.16	82.9	58.4	101.4	1.3	0.1	58.4	2.1	0.2	101.4
110	0.16	1.5	59.0	51.0	9.1	0.09	0.9	51.0	0.07	0.7	9.1

**Figure 4:**
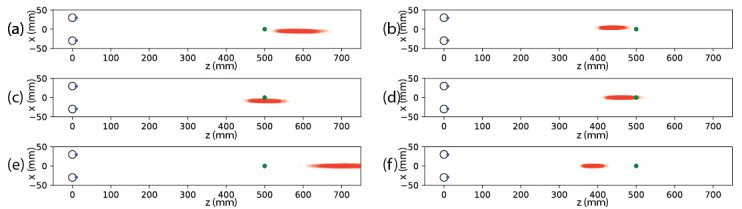
Effects of systematic offsets (inaccuracy) on the distribution of the vergence estimations. The distance between the two eyes is 6 cm and the object is at a distance of 50 cm. Each eye ray is distorted with zero-mean Gaussian noise with standard deviation of 0.16 degree. In the plots, we see the effects when a systematic offset (inaccuracy) of 1 degree is applied, not an uncommon effect size from a small change in pupil dilation. (a) Left eye is horizontally rotated by one degree outwards. (b) Left eye is horizontally rotated towards the right eye direction by one degree (similar distributions are obtained when right eye is horizontally rotated left by one degree). (c) Both left and right eyes are rotated in the same direction by one degree. Figures (d) and (e) show the distribution of vergence points when both eyes are systematically rotated inwards and outwards by one degree respectively. Systematic offsets in vertical direction lead to larger estimation errors without obvious shifts of the distribution mean, except when the offsets in opposite vertical directions are applied as shown in (f). Similar to the previous simulation of variable error (noise), vertical offsets lead to a bias towards the observer, i.e. the estimated mean vergence point is closer to the observer than the target.

Secondly, although it is not the focus of this paper, our noise formulation could also be used to investigate the effect of systematic errors (inaccuracy, offset) by introducing constant offset for the eye positions in Equation 12. As shown in the simulation results in Figure 4, unsurprisingly, the resulting errors, i.e. the distances between the estimated mean vergence point and the true target point, are larger when inaccuracy is introduced compared to when only noise is added to data. Furthermore, systematic offsets in the horizontal direction shift the mean of the estimation no matter whether the offsets in the two eyes converge or diverge. Meanwhile, vertical systematic offsets in the same direction lead to larger estimation errors without shifting the distribution mean much. Again, the estimated mean is biased towards the observer when systematic offsets in opposite vertical directions are introduced, similar to the bias we observed when only noise was introduced where the estimation of the mean vergence point is always closer to the observer than the true target point.

### Qualitative analytical analysis of bias

In the section above (and examples shown in Figure 3), we use a simple numerical simulation to visualize the distribution of computed points of interest given an assumption on the probability distribution of the noise. This simulation suggested that the mean vergence point is biased, shifted away from the true target point, depending on the standard deviation of the noise distribution in horizontal vs. vertical directions. We wish to analyze this behavior analytically. For this we attempt to compute the mean (or expected) vergence point for a given probability distribution of noise. As we will see, the general problem is difficult to approach. Yet, by assuming the geometric situation exhibits symmetry (see Appendix A for details) we are able to show that the trend we have observed in the numerical simulation holds qualitatively for wide classes of practically relevant scenarios.

The expected value for a discrete noise distribution would be the sum of values multiplied with the respective probabilities for the input parameters. In the continuous case, this sum turns into an integral over the product of the computed value and the probability distribution for the input parameters.

The projectors ${E^{\prime }}_{l,r}\left(\varepsilon_{l,r}\right)$ are generated from the noisy eye rays ${e^{\prime }}_{l,r}$. Assuming a probability distribution ${p^{\prime }}_{l,r}\left(\varepsilon_{l,r}\right)$ the expected intersection point is:

(16) $$x=\iint {\left({E^{\prime }}_{l}(\varepsilon_{l})+{E^{\prime }}_{r}(\varepsilon_{r})\right)}^{-1}\left({E^{\prime }}_{l}(\varepsilon_{l})p_{l}+{E^{\prime }}_{r}(\varepsilon_{r})p_{r}\right)p_{l}(\varepsilon_{l})p_{r}(\varepsilon_{r})d\varepsilon_{l}d\varepsilon_{r}$$

This integral, in general, cannot be treated analytically. However, note that the resulting mean is a linear function of the individual points of vergence. This means we can generate the mean of several instances of the distribution and then integrate over these local means. We will make use of this property in the following.

From the perspective of applications, we are mostly interested in understanding the bias with respect to different distances in depth. This allows us to concentrate on geometric configurations that exhibit symmetry. First, we assume the fixated object is on the symmetry line and at unit distance, i.e. ***z*** = (0, 0, 1)*^T^*. Note that by setting the interocular distance ***p****_r_* − ***p****_r_* = 2*a* this still considers arbitrary distances because geometrically it makes no difference if we change the distance of the target or the distance between the eyes. Second, we assume the probability distributions for left and right eyes are identical. We denote this by dropping the subscript: *p* = ***p****_l_* = ***p****_r_*. Third, we may additionally assume the probability distributions are symmetric around the origin. Point symmetry of the distribution means the probabilities of the variable errors +*ε* and −*ε* are the same: *p*(+*ε*) = *p*(−*ε*). This would allow us to consider pairs of points based on the error vectors *ε_l_*, *ε_r_* and −*ε_l_*, −*ε_r_*, which are symmetric around the line (0, 0, 1)*^T^* (i.e. one error vector is the reflection of the other by the line). Since their probabilities are the same, their mean is on this line. Pairing all instances of variable errors in this way shows that the mean would be on the symmetry line, for all probability distributions with point symmetry.

**Figure 5:**
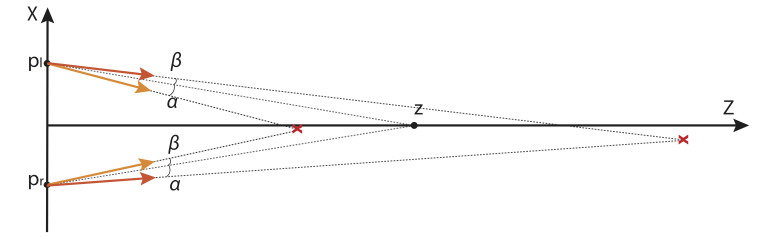
Illustration of two intersection points with inverted pair of errors *α*, *β*. Inverted pair of errors leads to an intersection point in the opposite directions; however, the mean vergence point is biased to be further away from the target point ***z***. In this illustration, *α* = 5° and *β* = −3°, where positive angle corresponds to the clockwise direction.

Consider the pair of error vectors *ε_l_*, *ε_r_* and the reversed pair *ε_l_*, *ε_r_*. By our assumptions, these instances have the same probability. The two pairs give rise to two points of interest. Figure 5 illustrates the case of horizontal error only. In this case, one of the two intersection points is closer to the observer then the target, while the other one further away. Their mean will never be closer then the real target, indicating that the mean over all instances with horizontal noise only will be biased to be further away than the target – as observed in the numerical simulations. We now prove that this intuitive reasoning is correct.

To compute the expected depth value, we start by analytically solving the least squares estimation (LSE) in Eq. 10 for the last component. This can be done by elementary computations. In this way we can express the depth for a certain pair of error vectors *ε_l_*, *ε_r_* as well as for the reversed pair *ε_l_*, *ε_r_*. Then we take the mean of the two depth values. All computations can be carried out by a computer algebra system such as Maple or Mathematica. The resulting expression is lengthy and unsuitable for direct inspection. However, considering the case of horizontal noise only leads to the following simple expression:

(17) $$\frac{z^{\prime }}{z}=\frac{4a^{2}}{4a^{2}-{\left(\eta_{l}-\eta_{r}\right)}^{2}}$$

As defined in Equation 1 and 12, 2*a* equals to the interocular distance and *η_l_*_/_*_r_* represents the horizontal component of variable error *ε_l_*_/_*_r_*. It makes sense to assume that the distance between the displacements *η_l_*_/_*_r_* is smaller than the interocular distance 2*a* (otherwise the intersection of the eye rays would be behind the observer). In this case the denominator is positive but never larger than the numerator, so the depth is larger. Because this is true for any pair of instances (see more in Appendix A), this is also true for the mean resulting from an arbitrary probability distribution (as long as they are they same for both eyes). This confirms our intuitive geometric conclusion from Figure 5.

Next we consider only vertical noise *ν_l_*_/_*_r_*. In this case it is convenient to consider the pair of error vectors *ε_l_*, *ε_r_* and −*ε_l_*, −*ε_r_*. Note that compared to the former case we now also reverse the signs, which is admissible if the probability distribution is symmetric. Using computer algebra as before, we find that the relative mean depth for the two intersection points is

(18) $$\frac{z^{\prime }}{z}=\frac{4a^{2}+a^{2}{\left(v_{l}+v_{r}\right)}^{2}}{{\left(v_{l}-v_{r}\right)}^{2}+4a^{2}+a^{2}{\left(v_{l}+v_{r}\right)}^{2}}.$$

Comparing numerator and denominator reveals that *z*′ ≤ *z* with equality only for *ν_l_* = *ν_r_* regardless of the probability distribution and without any restrictions on the vertical noise.

Together, these two results confirm our observations that noise in horizontal direction biases the mean depth towards larger values, while noise in vertical direction biases it towards the observer. This result holds for all probability distributions, as long as they are identical for both eyes (and exhibit symmetry in the vertical direction).

In any realistic scenario, however, noise errors have horizontal and vertical components. The resulting bias will depend on the relative magnitude of these errors. If the magnitude of horizontal and vertical noise error is equal, then the bias is toward smaller depth and the magnitude of this effect is dominated by the squared difference (*ν_l_* − *ν_r_*)^2^. In general, however, the variance along the horizontal versus vertical axes in the probability distribution determines the bias in depth. If the variance is higher in horizontal direction, the depth will be biased towards greater values; if the variance is higher in vertical direction, depth will be biased closer to the viewer. As before, this result holds for probability distributions as long as they are identical for both eyes and the point closest to any two eye rays in the distributions is in front of the viewer (not behind).

## Part 2: Human data

The numerical simulations above show that variable and systematic errors in eye rays have a significant influence on the estimated mean point of vergence under projection. In this section, we collect human vergence data to study the real noise distributions when using a video-based eye-tracker. Eye-trackers primarily output mapped gaze positions, which in 2D are points on a screen, described in pixel coordinates. This mapping is established using a calibration routine, where participants are asked to look at several targets on the screen while features of their eyes are tracked in the camera image. These pre-calibrated features - the pupil centre and the centre of the reflection in the cornea - are mapped to gaze positions on the monitor during calibration. With an established mapping, any eye positions can be mapped into the target space, which corresponds to the gaze point.

Several sources of errors are known to interfere in this procedure, in particular when both eyes are being calibrated. Despite existing research on vergence eye movements [2, 3, 21], there is no established consensus on how to calibrate binocularly. [5] have brought up the question whether binocular calibration (i.e. calibrating both eyes at the same time) or monocular calibration (i.e. calibrate one eye at one time while the view of the other eye is occluded) is better suited for binocular eye movements study. Besides the intrinsic properties of eye movements in binocular viewing, the estimated mapping function in calibration also introduces errors in the estimated point of interest. The estimation of parameters in the mapping function is a minimization procedure. In practice, low order polynomials are often used to map pupil and corneal reflection positions onto screen coordinates, and the mapping parameters are approximated through an optimization procedure (e.g. least squares), which inevitably contains modeling errors.

Therefore, we designed a data collection including both binocular and monocular viewing conditions and analyzed the detected raw pupil and corneal reflection positions without mapping them to the target space. Two depth variations were included and we used symmetric presentation of stimuli to counterbalance any potential behavioral differences due to spatial dependency.

### Participants

25 participants from TU Berlin (students and staff) joined our experiment (mean age = 27, SD = 7, 4 females). They all had normal or corrected to normal vision and provided informed consent. Participants with glasses were excluded from the experiment due to concerns over eye tracking accuracy. Five of them had previous experience with eye tracking experiments. Their time was compensated. Additionally we performed a monocular eye examination for each participants and the averaged acuity is 0.93 (SD=0.26) measured in the decimal system. We also measured the eye dominance of observers following the standard sighting eye dominance test, as our experiment does not involve any interocular conflict. Observers were asked to look at a distant point though a small hole placed at arm length, and then to close their eye one after another. Only the dominant eye supposes to see the point while viewing monocularly. Among all 25 observers, only 7 of them has a left dominant eye while 18 of them reported to have a right dominant eye. We discuss on the eye dominance test in the discussion section.

### Apparatus and recording setup

The data collection was conducted in a quiet and dark room. We used an EyeLink 1000 desktop mount system in the experiment and binocular eye movements were tracked at a sampling rate of 1000 Hz. A chin-forehead rest was used to stabilize observers’ head positions. A 24-inch display (0.52 m × 0.32 m, 1920 × 1200 pixels) was placed at two distances of 0.7 m and 1.1 m from the eyes. Stimulus presentation was controlled using PyLink provided by SR Research. Note that any eye trackers can be used for the recordings as our analysis is based on detected eye positions in the camera frames.

Two custom-built eye covers fabricated by 3D printing were mounted on the chin-forehead rest (see Figure on the right). Each of them can be rotated by 180° to open or block the view of one eye.

**Figure:**
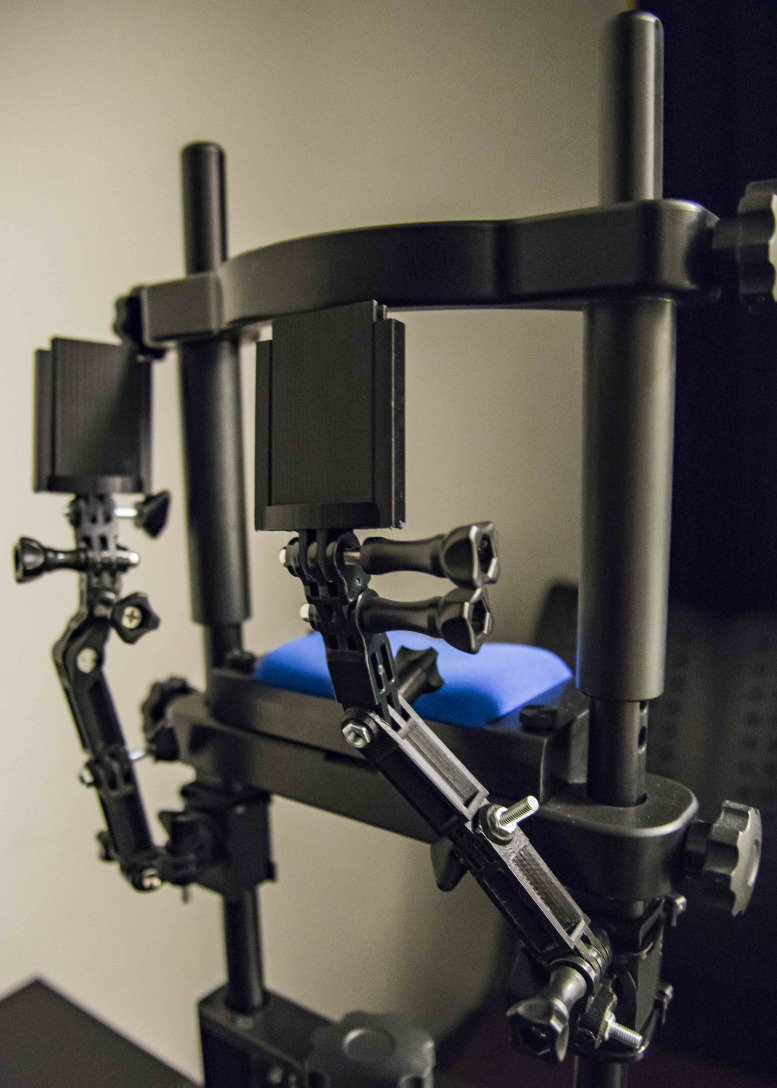


### Design and procedure

In order to familiarize observers with fixational eye movements, and to verify the setup that observers’ eyes were clearly tracked, we ran a default 9-point calibration routine before the data collection started. A calibration is followed by a validation to verify its accuracy. Experiment was continued only when the achieved validation accuracy is on average smaller than 1.0° of visual angle. Otherwise we either repeated the calibration and validation routine immediately or after a short break. However, the resulting gaze data are not used. All further analyses are purely based on the pre-calibrated pupil minus CR (corneal reflection) data, to minimize influence from the calibration mathematics of the EyeLink.

The data collection consisted of two distances and each distance had five repetitions. In each repetition, we presented three viewing conditions, namely monocular viewing with left eye, monocular viewing with right eye, and binocular viewing. Eye covers were rotated by the instructor to occlude/reveal the eyes in different viewing conditions. Meanwhile, the EyeLink was continuously tracking in binocular mode throughout.

**Figure 6:**
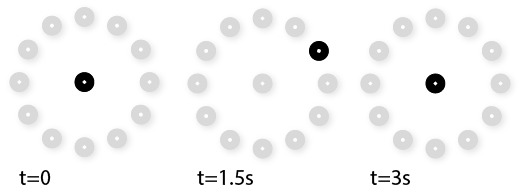
An exemplary trial. The center was always presented at the beginning of each trial. A randomly selected marker on the ring was then presented for 1.5s followed by the presentation of the center marker for another 1.5s. Then another random selected marker on the ring was presented. The presentation continues in such way until all markers on the ring were presented once. Gray markers are drawn here for illustration purpose and were invisible during experiment.

For each distance, repetition and condition, the participant was asked to fixate each one out of 12 targets in the form of a ring with an addition central point (Figure 6). Participants were instructed to follow the marker and fixate the white dot at the center as accurately as possible. Black circles with their center marked by a white dot were used as the target. The radius of the ring corresponded to 7° of visual angle. Each marker was presented for 1.5 s and a beep sound was used to signal the start. Between each fixation trial, participants refixated the center point. In total, there were 12 targets fixations on the periphery targets and 12 at the center target.

Each block of five repetitions for a distance took about 20 minutes and participants were asked to take a break before the second block. In total it lasted about one hour including a simple trial run at the beginning.

### Analysis Methods and Results

Our analysis was based on raw samples of detected eye positions, i.e., the pupil minus CR positions, represented as pixel coordinates in the eye camera. The only use of calibrated gaze was our use of the built-in velocity-based algorithm of the EyeLink software to detect fixations. In order to find fixations on targets, we filtered out all short fixations that are less than 100ms in a preprocessing step as short fixations very often are the result of undershoots ([26], p.222-223) and are quickly followed by a correction saccade onto targets. Conservatively, among fixations on the same target, we considered those that are two standard deviations away from the cluster center as outliers. The remaining fixations were from then on only processed as uncalibrated pupil minus CR.

For each dataset of one observer, we aggregated all fixations (pupil-CR) in the five repetitions for each target in the same viewing condition. On average there are 56 fixations in each such block (SD = 20), and each target has 2.3 fixations as we have 24 fixation targets in each repetition. Here fixations from individual eye are considered independently. The averaged duration of fixations is 821ms (SD = 494ms). There is no significant difference among different viewing conditions at two distances.

### Comparing sets of covariance matrices

In the previous simulation (see Section “Ray Errors”), fixation positions have independent zero-mean Gaussian distributions for variable errors in horizontal and vertical directions, which can be represented by a 2 × 2 diagonal covariance matrix Σ. Diagonal elements in Σ describe the variances in horizontal and vertical directions separately and off-diagonal elements show the correlation between them. Therefore, Σ is a positive semi-definite symmetric matrix, and it can be visualized as an error ellipse with its axes pointing into the directions of its eigenvectors. The lengths of the semi-axes are proportional to the square roots of the corresponding eigenvalues *λ_i_*. We used $\sqrt{5.991}\sqrt{\lambda_{i}}$ as the semi-axis length (derived from a Chi-Square distribution), which gave us the ellipse that covers a 95% confidence interval. We continued using covariance matrices to analyze the error distributions and visualized them as ellipses in the following. Essentially covariance matrix measures the precision in two dimensions, as we are interested in its special distribution.

In our data collection, we had a set of markers on screen and a covariance matrix represented fixation distribution at each marker position. To model the variability among different covariance matrices and to compare the differences among sets of covariance matrices, we computed distances between all pairs of covariance matrices in a set and then compared the resulting distributions. Despite the raising amount of applications of analyzing the variance among covariance matrices, there is still no consensus on how to analyze the covariance structures [27]. We settled on a logarithm-based distance estimator [28], a Riemannian metric, defined as

(19) $$d(\Sigma_{1},\;\Sigma_{2})=\left\|\log(\Sigma_{1}^{-\frac{1}{2}}\Sigma_{2}\Sigma_{1}^{-\frac{1}{2}})\right\|,$$

where the logarithm is given by *log* Σ = ***U**** log* (***S***)***V*** and ***U***, ***S***, ***V*** can be factorized from singular-value decomposition (SVD) as Σ = ***USV***. ***S*** is a diagonal matrix of singular values of Σ. ∥***X***∥ is the Euclidean norm (also called Frobenius norm) and it can be computed by the matrix trace $∥X∥\;=\;\sqrt{Tr\left(X^{T}X\right)}.$ In case of covariance matrix, the trace measures the total variation in each dimension without considering the correlations among variables. As shown in [28], this distance measure is symmetric and non-negative, and it is invariant under affine transformation and inversion.

Intuitively speaking, by multiplying with ${\text{Σ}}_{1}^{-\frac{1}{2}}$ in bilinear form, we transform Σ_2_ into a new basis $\Sigma_{1}^{-\frac{1}{2}}\;\Sigma_{2}\;\Sigma_{1}^{-\frac{1}{2}}$ is a perfect circle. The distance measures the relative ratio of eigenvalues in the new basis and the largest eigenvalue of ${\text{Σ}}_{1}^{-\frac{1}{2}}\;\Sigma_{2}\;{\text{Σ}}_{1}^{-\frac{1}{2}}$ corresponds to the ratio of maximum variance between two groups [27]. To aggregate a set of covariance matrices, we used the arithmetic average as the mean covariance matrix. [29] compared many covariance distance measures especially in the context of shape interpolation; however, it is not clear which one suits best in our case. Here we only want to be able to compare sets of covariance matrices.

In the next step, the distance distributions of two sets of covariance matrices were compared using the Kolmogorov-Smirnov test (KS-test) [30] and a p-value was computed to determine whether the two distributions differ significantly. The KS-test computes the vertical distance between two cumulative fraction functions that are used to represent two distributions and takes the largest distance as the statistic. Therefore, it is robust with respect to variance types of distributions.

### Results

Below, we examined whether the bias was present also in the human data. We first compared the distributions of variable errors among all individual observers. Then we aggregated all datasets to examine whether the distributions has any spatial dependency. In the last step, we used the averaged variable error distribution to sample eye positions following the procedure in the previous simulation (see Section Ray Error) to investigate whether there is a bias in the direction of the observer in human data as there was in simulated data.

#### Eye dominance and acuity do not seem to matter

**Figure 7:**
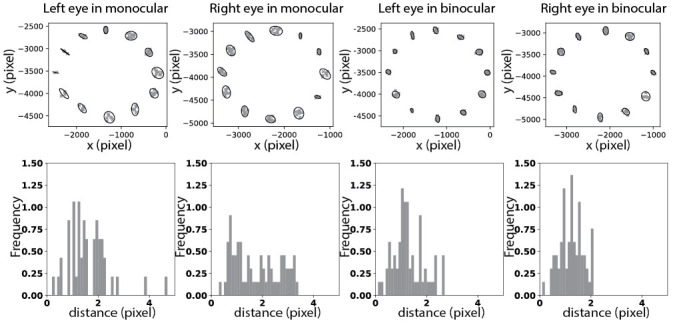
Example of one-dataset samples at the distance of 70 cm. In the first row, fixations at each marker position collected over five repeats are scattered and corresponding covariance matrices are visualized as ellipses. Second row shows the histograms of distances between all pairs of ellipses in each condition (summed over five repeats). Observer of this dataset has right dominant eye and eye acuity is 0.9 for left eye and 1.0 for right eye.

Each dataset (of one observer) had five repetitions of each viewing condition. Each eye had two viewing conditions, namely monocular viewing and binocular viewing. Targets were presented at two different distances. We first aligned the five repetitions in the same condition and computed the covariance matrix of fixations at each marker position. Following Equation 18, each covariance matrix was compared to the other eleven covariance matrices. Note that we discarded all fixations at the center marker. One exemplar dataset is shown in Figure 7. Covariance matrices are visualized as ellipses and distributions of all pair-wise distances are plotted as histograms.

**Figure 8:**
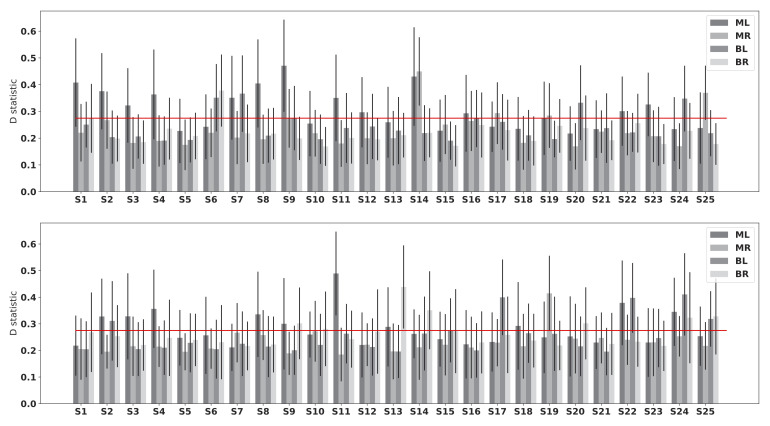
Statistics of KS-test on individual differences. Upper plot shows the statistics of data with a display placed at a distance of 70 cm and the lower one shows the result when the display was at a distance of 110 cm. Red lines mark the 0.05 significance level. ML stands for the condition of monocular viewing of left eye, MR monocular viewing of right eye, BL binocular viewing of left eye and BR binocular viewing of right eye.

Using the KS-test, we compared histograms of the distance distributions of each individual dataset to the others and results are shown in Figure 8. Red line marks the significance level of 0.05. Note that any statistic value larger than the level (i.e. above the line) corresponds to a significantly different distribution. Considering each eye separately, we had four viewing conditions in one dataset, namely monocular viewing of left eye (ML), monocular viewing of right eye (MR), binocular viewing of left eye (BL) and binocular viewing of right eye (BR). At the distance of 70 cm, 13 out of 25 datasets have significantly different fixation distributions in ML, 4 in MR, 5 in BL and 1 in BR. According to the categorization of eye dominance, we have 8 significantly different distributions of dominant eye in monocular viewing and 12 significantly different distributions of nondominant eye. In binocular viewing condition, 2 distributions of dominant eye significantly differ from the others and 6 of nondominant eye. At the distance of 110 cm, the numbers of datasets, which are significantly different from the others, are 10 (ML), 1 (MR), 6 (BL), and 7 (BR). In monocular viewing, 3 and 9 datasets are significantly different for distributions of dominant eye and nondominant eye respectively. In binocular viewing condition, the number of different datasets is 9 for dominant eye and 6 for nondominant eye. Note that this counting is based on the mean statistic value. And we do observer large variances in each dataset as shown in Figure 8.

In conclusion, the differences between dominant and non-dominant eye are so small and varied that we cannot conclude that eye dominance matters. Neither did we observe any correlation between the distribution differences and eye acuity, which indicates that eye acuity does not contribute to the differences in distributions. It is likely that other factors, such as eye colour, may explain part of the noise.

#### Noise does not vary depending on where participants look

**Figure 9:**
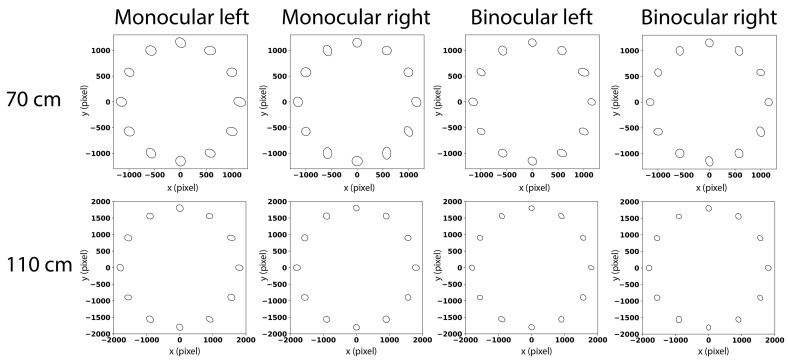
Visualization of mean covariance matrices at each marker position. First row shows error distributions of data collected at a distance of 70 cm and second row shows data collected at a distance of 110 cm. In each viewing condition at one distance, data from all observers were aggregated together and covariances at each marker position are visualized as ellipses. For instance, a left-tilted ellipse means that vertical noise is larger than horizontal noise. The size of the ellipse thus corresponds to the total variation while orientation indicates the correlation between horizontal and vertical directions.

It is known that noise varies across the measurement space ([26], p.182). To test whether there is any such spatial dependency of noise distributions of fixations, we computed the distance distribution at each marker position by comparing each pair of covariance matrices from 25 datasets. Similarly, we applied the KS-test to compare the distance distributions. Only one distribution from monocular viewing of right eye when screen was at a distance of 70 cm is significantly different. To visualize the variance at each marker position, we computed the mean of 25 covariance matrices for each marker in one viewing condition and plotted corresponding ellipses in Figure 9. Note that comparing two distances, covariance ellipses are similar in both sizes and orientations. But ellipses in binocular viewing condition have smaller sizes but still similar orientations. In conclusion, the amount of noise does not seem to differ between the conditions, nor between positions. There is however a tendency that vertical noise is larger than horizontal noise.

#### But there is bias in the human data

After we have established that noise does not vary across eye dominance, acuity and fixation position, we can now aggregate the covariance matrices at all marker positions, which gives us an overall representation of noise distribution of fixations in each condition. Using the distributions - a covariance matrix for each of the 12 fixation point in each condition - rather than the data themselves introduces no bias, but is computationally easier, since it allows us to calculate the mean easily without being biased by unevenly taken data (i.e. uneven contribution of individual observers because of fixations of differing lengths).

**Figure 10:**
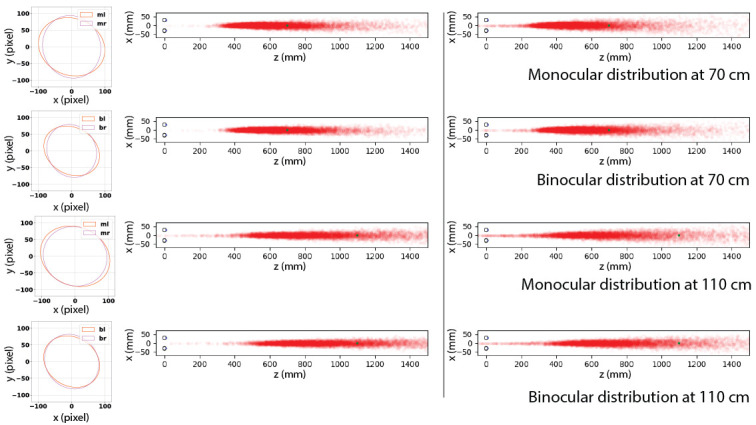
Estimation of mean vergence point based on distributions formed from real human data. Left column shows the mean covariance matrices of left eye (l) and right eye (r) in monocular viewing (m) and binocular viewing (b). First two rows show results when target is placed at a distance of 70 cm and last two rows show results at a distance of 110 cm. Right column shows the distribution of estimated vergence points in space where variance (visualized in the left column) was converted to 1° and 2° of visual angle respectively. See Table 2 for more detailed statistics.

We used the arithmetic mean of the 12 covariance matrices (of 12 targets) to represent the fixation distribution in each viewing condition and obtained two covariance matrices, one for each eye in either monocular or binocular viewing condition at one distance. As shown in Figure 10, an ellipse is used to represent a covariance matrix. Errors seem to be larger when targets move from 70 cm to 110 cm with increased sizes of ellipses. Noise in binocular viewing condition is smaller comparing to that in monocular viewing condition, evidenced by smaller ellipses in most positions in all conditions. The radius of the ring corresponds to 7° of visual angle and opposite sample positions span a visual angle of 14°. Following this ratio, we converted sample units (measured in pixel) into degrees of visual angle and applied the analysis framework used in the simulation above. Variances of error distributions shown in Figure 10 approximate to 1° of visual angle, which is commonly used as a calibration threshold in 2D eye tracking experiment. Additionally we also experiment with 2° of visual angle, which is equivalent to the start-of-art eye tracking accuracy in 3D space [9, 10, 13]. Simulated results based on sampling from real error distributions are plotted in Figure 10. On the left side we have spatial distributions of vergence points when variance was converted to 1° of visual angle and on the right side we see the results after converting to 2° of visual angle. Detailed statistic results are given in Table 2. Although the error in estimated mean vergence point is acceptable when everything is perfect within 1° of visual angle, however, achieving such accuracy in 3D is rather challenging. Even with an acceptable accuracy of 2°, there exists a strong bias towards the viewer in the estimated mean point of vergence.

**Table 2:** Vergence errors sampled from real noise distributions. σ represents converted standard deviation in degree of visual angle. ${\bar{\text{p}}}_{\text{z}}$ represents the mean point of vergence in depth measured in cm. Averaged errors and standard deviations are reported in cm with x represents the horizontal direction, y the vertical direction, and z in depth. On average, the errors in percentage of the corresponding distance are 30% (σ = 1) and 46% (*σ* = 2) when target distance is 70 cm, and 41% (*σ* = 1) and 50% (*σ* = 2) when target distance is 110 cm.

target distance (cm)	*σ* (deg)	condition	${\bar{p}}_{z}$ (cm)	$\bar{error}$ (cm)	$\bar{std}$ (cm)	${\bar{error}}_{x}$ (cm)	${\bar{error}}_{y}$ (cm)	${\bar{error}}_{z}$ (cm)	${\bar{std}}_{x}$ (cm)	${\bar{std}}_{y}$ (cm)	${\bar{std}}_{z}$ (cm)
70	1.0	monocular	69.1	22.4	32.0	0.88	0.86	22.3	0.88	0.90	32.0
		binocular	70.4	18.9	27.8	0.76	0.74	18.8	0.79	0.77	27.8
	2.0	monocular	60.0	35.1	82.6	1.2	1.2	34.9	2.8	2.1	82.6
		binocular	64.3	28.6	53.7	1.1	1.0	28.5	1.3	1.3	53.8
110	1.0	monocular	98.5	49.9	102.3	1.2	1.1	49.8	1.5	1.7	102.3
		binocular	104.4	41.6	88.8	9.9	9.5	41.5	1.4	1.2	88.8
	2.0	monocular	73.3	70.1	141.2	1.5	1.3	70.0	2.5	2.8	141.2
		binocular	86.7	60.2	171.7	1.3	1.3	60.1	2.3	2.7	171.7

## Minimizing the uncertainty in vergence point estimation

When researching vergence using a video-based pupil and corneal reflection eye-tracker such as the Eyelink, what can you do to minimize biases and errors? We assume that the human participant has a negligible difference in gaze directions between left and right eye, that luminance conditions are fixed and no other effects on pupil dilation are present, and that the only remaining issue is to minimize the bias from the noise in the signal.

As this bias is an effect of the projective mapping, the non-linear mapping that is commonly used to estimate the vergence point in space, there is not much you can do if you use the calibration routine shipped with the eye- tracker. The single fixation per calibration point will have an inaccuracy and noise that increases the bias. However, it is possible to instead record multiple fixations on each *point in your own set of c*alibration targets. Then take the average of the data samples in the several fixations per fixation target and use that average to calibrate the eye-tracker. The key is to use many fixations per calibration point. The same principle applies to real experiment after calibration: collect many fixations if possible. For each point of interest, taking the average of fixation samples in the camera space leads to better estimation in space. In practice, this could mean a repetition of the same experimental condition, for example, where observers are asked to refixate on the same targets in a vergence study.

**Figure 11:**
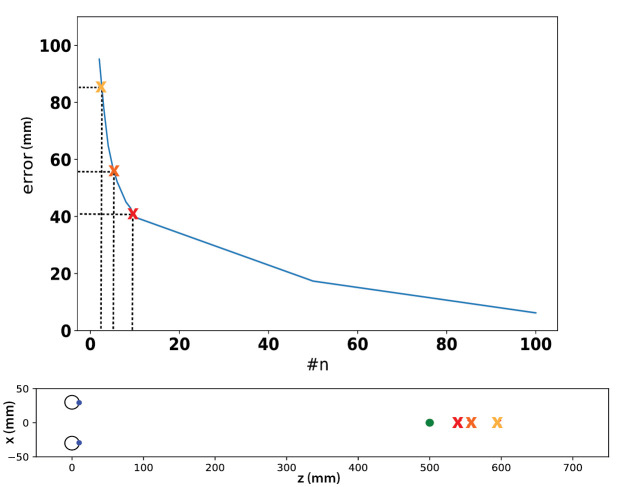
More fixations per target leads to better estimation of mean vergence point. At the top of the figure, offset error is plotted as a function of number of fixations per calibration point. Three color-coded crosses mark the offset errors when number of fixations equal to 2, 5, and 10. These three offsets are visualized in a top view plot at the bottom. We used standard deviation of 1.5 in horizontal direction and 0.16 in vertical direction.

The reason this method works is that for geometrical reasons, it is better to average in the calibration data than in the depth data of the intersection points. Assume that noise in the eye samples are Gaussian distributed, taking the average in the calibration data leads to better approximation to the noise-free eye samples. However, due to the non-linearity of the mapping, averaging in the depth data only leads to a bias as we see before. Figure 11 shows for simulated data how the offset in depth decreases with an increasing number of fixations per calibration point.

This solution works on the DPI, the EyeLink and the SMI eye-trackers, which all provide access to the center point of the corneal reflection and the pupil (for EyeLink and SMI) or the 4th Purkinje (for the DPI) in the data. Note that the recipe works with raw positions of the eyes and does not depend on specific calibration model given by any eye trackers.

## Discussion

The major finding in this paper is that vergence data from eye-trackers exhibit a bias, depending on the noise level in horizontal vs. vertical directions. This applies to the estimation of the point of vergence in three-dimensional space, as well as to the vergence estimation on planar surface in two-dimensional space. As long as a projective model is used in the mapping, the estimated mean vergence point will be biased.

### Reading research and calibration algorithms

The bias we have found is in line with [5], who reported that the fixations during reading were almost always crossed, peaking 2.6 cm in front of the plane of text, which is very much in line with the error reported in Table 2. However, instead of resulting in a discussion about potential biases from the measurement technology itself, the subsequent papers instead investigated whether monocular vs. binocular calibration could cause the crossing of fixations.

Liversedge et al.[31] had found that “When the points of fixation were disparate, the lines of gaze were generally diverged (uncrossed) relative to the text (93% of fixations),..., but occasionally converged (crossed) (7% of fixations).” using a monocular calibration. Later study by Kirkby et al. [32] replicated the finding that - after monocular calibration - the majority of fixations are uncrossed, both with the EyeLink and the DPI.

Our results using the collected human data show a significant bias towards observers for both monocular and binocular calibration, corresponding to crossed fixations in reading. This bias must have existed also for the studies by Liversedge et al. [31] and Kirkby et al. [32], so it is surprising that they found crossing result in the opposite direction of the bias.

Švede et al. [33] argued that monocular calibration is the only physiologically correct form in the sense that it preserves the difference in gaze direction between the two eyes. It could be the case that the average gaze differences between left and right eye after monocular calibration are so large that they consume the whole bias and nevertheless can remain uncrossed.

Despite the fact that the bias in the estimated gaze positions in binocular viewing is smaller than the bias in monocular viewing condition, the ratio in depth between target position and the estimated mean vergence point is around 1.2.

### Eye dominance, interpupillary distance and fixation disparity

The collected human data show a large variance among individual observers. However, neither eye dominance nor acuity leads to significantly different noise distribution and where participants look also doesn’t seem to matter. Nevertheless we should be aware that eye dominance information is based on observer’s self-report. Moreover, the eye dominance is determined by the so-called sighting eye dominance test. A recent study [34] brought up the question whether one type of eye dominance exists and their results indicate disagreements among different eye dominance test methods, especially the difference between sighting eye dominance test and binocular rivalry based test. Even though our experiment does not involve any interocular conflict, the actual dominant eye during the experiment might still be different from the measured one, which might further explain the observed no-impact findings.

Interpupillary distances may also influence the bias, as it changes the geometry, i.e. the short edge of the triangle formed by the two eyes and a target. How the bias is correlated to the interpupillary distance is not studied in this work.

It is not clear so far how fixation disparity may contribute to our observations. [4] reported that fixation disparity decreased over time during reading, and it is tightly linked to vergence eye movements [35, 36, 37] as well as binocular vision [38]. Additionally, vision training may improve stereo perception and eliminate fixation disparity [39]. We neither performed any fixation disparity test nor measured the binocular version in our study, although how to accurately measure fixation disparity seems to be an on-going effort [40]. Future studies should include measurements such as the near point of convergence, positive and negative fusional range, dissociated phoria at near and far, stereopsis and amplitude of accommodation. It would also be interesting to test whether there exists a correlation between fixation disparity and the bias in individual’s dataset.

### Limitations of our study

Independent Gaussian distributions were used for each dimension. It would be interesting to study the dependency between noise in horizontal and vertical directions and to see how a multivariate distribution influences the bias.

In our experiment targets were displayed on a flat plane without the need of focus change. But noise distribution might be different due to the dynamic changes of focus in 3D. For example we might need to take into account the changes of pupil size. How to collect enough fixation data in 3D while maintaining the same precision is another practical but challenging problem.

To minimize the bias from the signal noise, we suggest to collect many fixations per calibration target, and then use the mean fixation point to calculate the mapping. Future work should validate this proposal with real human data. Note that this proposed recipe does not account for fixation disparity, i.e. the alignment difference between the dominant eye and the weak eye.

### Suggestion for future experiments

There seems to be no good reason to prefer one viewing condition to the other in calibration. In monocular calibration, each individual eye is forced to fixate on targets without the interference of binocular fusion and eye dominance. However, visual acuity is also limited in monocular viewing and possibly decreases over distances. Precision of eye movements in binocular viewing condition seems to be higher.

We believe it is important to be aware that the propagation of noise may lead to a bias in the estimated mean vergence point in space. It is also very important for future eye movement studies, especially for vergence studies, to provide the validation error of calibration not just as a single scalar, but also in the form of a spatial distribution (i.e. an error ellipse). It provides a confidence level of the observed data and puts their interpretation into perspective. It is not even clear if the error distribution would be roughly similar for all types of tracking devices and experiments, or if the distribution changed with the type of experimental task. If so, resulting mean vergence depth would vary with experimental setup.

Moreover, our results suggest that, for researchers using eye-tracking devices, it is good to think about the procedure, instead of being only concerned about the data after calibration. The mathematical models behind calibration might provide additional information as being part of the experiment.

## Conclusions

The estimated point of interest from intersection of eye rays has large error in depth. The mean of the linearly estimated points of vergence is biased and depends on the horizontal vs. vertical noise distribution of the fixation positions. It is generally hard to interpret results for depth from binocular vision and our suggestion is to take the average of fixations of the same target to minimize the uncertainty, in both calibration and experiment phase.

## Ethics and Conflict of Interest

The authors declare that the contents of the article are in agreement with the ethics described in http://biblio.unibe.ch/portale/elibrary/BOP/jemr/ethics.html and that there is no conflict of interest regarding the publication of this paper.

## Figures and Tables

**Figure 12:**
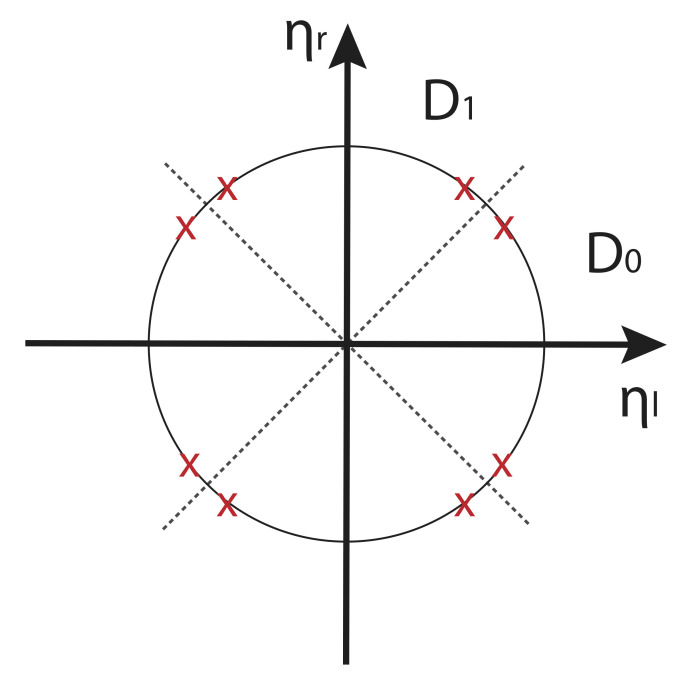
Symmetry of error pairs. x and y axes correspond to the horizontal errors of left eye *η_l_* and right eye *η_r_*. Eight crosses on the circle have the same probability.

## Appendix A: Analytical analysis of bias

Our variable errors contain independent horizontal and vertical components. Each of them follows the zero-mean Gaussian distribution, which is a symmetric function around 0, i.e. *p*(*a*) = *p*(−*a*).

To simplify the scenario, we set vertical errors to zero and only consider errors in the horizontal direction. Assume left eye and right eye have identical Gaussian distributions, we have *p*(*η**_l_*, *η**_r_*) = *p*(*η**_r_*, *η**_l_*). The probability of observing one pair of errors is the same when it corresponds to left and right eyes or vice versa.

Based on previous two symmetries, we have the same probability for 8 error pairs as shown in Figure 12. As *η**_l_* and *η**_r_* follow two independent Gaussian distribution of the same variance, we have a joint normal distribution. Each circle centered at origin is a contour line where samples have the same probability. Given an error vector (*η**_l_*, *η**_r_*), we can find the other seven pairs by the following operations using symmetry:

– *p*(*η**_l_*, *η**_r_*) = *p*(−*η**_l_*, *η**_r_*) by changing the sign of left eye,
– *p*(*η**_l_*, *η**_r_*) = *p*(*η**_l_*, −*η**_r_*) by changing the sign of right eye,
– *p*(*η**_l_*, *η**_r_*) = *p*(−*η**_l_*, −*η**_r_*) by changing the sign of both eyes,
– *p*(*η**_l_*, *η**_r_*) = *p*(*η**_r_*, *η**_l_*) by swapping between left and right eyes,
– *p*(*η**_l_*, *η**_r_*) = *p*(−*η**_r_*, *η**_l_*) by swapping and changing one sign,
– *p*(*η**_l_*, *η**_r_*) = *p*(*η**_r_*, −*η**_l_*) by swapping and changing one sign,
– *p*(*η**_l_*, *η**_r_*) = *p*(−*η**_r_*, −*η**_l_*) by swapping and changing both signs.

Eight samples are symmetric across four lines which are *η**_l_* = 0, *η**_r_* = 0, *η**_l_* = *η**_r_*, *η**_l_* = −*η**_r_*. Together these four lines divide the whole variable domain into eight domains {*D**_i_* ,*i* = 0, ⋯, 7}. We define function Φ*_i_* that maps samples from *D*_0_ to *D*_1_. For example Φ_1_(*η**_l_*, *η**_r_*) ≔ (*η**_r_*, *η**_l_*) which maps sample in *D*_1_ to *D*_0_ as shown in Figure 12. As Φ*_i_* is a linear map, we know *det*(*d*Φ*_i_*) = *det*(Φ*_i_*) = 1.

Given a pair of horizontal errors (*η**_l_*, *η**_r_*), we know the intersection point x can be computed as

(20) $$x_{\eta_{l},\eta_{r}}={\left({E^{\prime }}_{l}(\eta_{l})+{E^{\prime }}_{r}(\eta_{r})\right)}^{-1}\left({E^{\prime }}_{l}(\eta_{l})p_{l}+{E^{\prime }}_{r}(\eta_{r})p_{r}\right)$$

and its probability is *p*(*η**_l_*, *η**_r_*) Let us define a function *f* that $f\left(\eta_{l}\;,\;\eta_{r}\right)∶=x_{\eta_{l}\;,\;\eta_{r}},\left(\eta_{l}\;,\;\eta_{r}\right)\in D_{i}$ and *f**_i_* = *f* ∘ Φ*_i_*. The expected value of *x* in the whole domain is

(21) $$\begin{array}{l} x=\iint f(\eta_{l},\eta_{r})p(\eta_{l},\eta_{r})d\eta_{l}d\eta_{r}\\ =\sum_{i=0}^{7}\iint_{D_{i}}f(\eta_{l},\eta_{r})p(\eta_{l},\eta_{r})d\eta_{l}d\eta_{r}\\ =\sum_{i=0}^{7}\iint_{\Phi_{i}(D_{0})}f(\eta_{l},\eta_{r})p(\eta_{l},\eta_{r})d\eta_{l}d\eta_{r}\\ =\sum_{i=0}^{7}\iint_{D_{0}}(f\circ \Phi_{i})(p\circ \Phi_{i})\det(d\Phi_{i})d\eta_{l}d\eta_{r}\\ =\sum_{i=0}^{7}\iint_{D_{0}}f_{i}pd\eta_{l}d\eta_{r}\\ =8\iint_{D_{0}}\sum_{i=0}^{7}\frac{1}{8}f_{i}pd\eta_{l}d\eta_{r} \end{array}$$

Since probability on the same contour line is the same, *p* ∘ Φ*_i_* = *p*. The expected intersection point *x* can be computed by computing the average of *f**_i_*. Therefore, we only show the simplified computation of one pair of error vectors in Section Qualitative analytical analysis of bias.

## Appendix B: Python script for simulation

**import** math
**import** numpy as np
# *little helper to generate normed vectors*
# *not safe to use with vanishing vectors!*

**def** normed(v):
     l = np. linalg .norm(v)
     **return** v/l

**def** closestpoint(p0,p1,ray0,ray1):
# *Generate projectors from rays*
     R0 = np.identity(3) − np.outer(ray0,ray0)
     R1 = np.identity(3) − np.outer(ray1,ray1)
     # *Solve linear system*
	 **return** np.linalg.solve(R0+R1,np.dot(R0,p0)+np.dot(R1,p1))

# *graphical output requires matplotlib*
graphical_output = True
# *we assume units are mm*
# *positions of left and right eye in the plane*
eye_l = np. array ((0.0 , −30.0 ,0.0))
eye_r = np. array ((0.0 ,30.0 ,0.0))
# *position of object*
lookat = np. array ((500.0 ,0.0 ,0.0))
# *direction between eyes*
eye_base = normed(eye_r – eye_l)
# *up direction*
# *should be orthogonal to eye_base*
up = np. array ((0.0 ,0.0 ,1.0))
# *normalized rays from eyes to object*
ray_l = normed(lookat – eye_l)
ray_r = normed( lookat – eye_r )
# *normalized horizontal and vertical direction per eye*
hor_l = normed(np.cross(ray_l ,up))
ver_l = np.cross(hor_l,ray_l)
hor_r = normed(np.cross(ray_r ,up))
ver_r = np.cross(hor_r,ray_r)
# *variance of the angular deviation*
# *we sample angular deviation on a tangent plane*
# *and then project back to the sphere*
variance_l = np. radians ([1.5 ,0.16])
variance_r = np. radians ([1.5 ,0.16])
#*print eyes and object*
**print**("Eyes at ",eye_l ," , ",eye_r)
# *containers to store x and y positions of*
# *intersection of left and right eye ray*
if graphical_output :
     x=[]
     y=[]
	 
min_psi=[]
 max_psi = []
max_s = 0.0
min_s = np.linalg.norm(0.5∗eye_r + 0.5∗eye_l-lookat)
# *number of random measurements*
trials = 10000
# *center of interest , i .e. average position*
coi = np.zeros(3)
**for** i **in** range(trials):
     # *change of angles*
     # *using normal distribution*
     psi_l = np.random.normal(0.0,variance_l)
     psi_r = np.random.normal(0.0,variance_r)
     # *using uniform distribution*
     #*psi_l = np.random.uniform(−2.0∗variance ,2.0∗variance)*
     #*psi_r = np.random.uniform(−2.0∗variance ,2.0∗variance)*
     # *using laplace distribution*
     #*psi_l = np.random.laplace(0.0,variance)*
     #*psi_r = np.random.laplace(0.0,variance)*
     # *generate the slightly rotated eye rays*
     # *simply by adding the components along eye and up direction*
     vray_l = normed(ray_l + psi_l[0] ∗ hor_l + psi_l[1] ∗ ver_l)
     vray_r = normed(ray_r + psi_r[0] ∗ hor_r + psi_r[1] ∗ ver_r)
     v = closestpoint(eye_l ,eye_r ,vray_l ,vray_r)
     # *keep track of centroid of estimated object positions*
     coi += v
     # *keep track of smallest and largest distance*
     # *we simply judge distance by length of s*
     ls = np.linalg.norm(0.5∗eye_r + 0.5∗eye_l − v)
	 **if** (ls>max_s):
          max_s = ls
          max_psi = [psi_l ,psi_r]
	 **if** (ls<min_s):
          min_s = ls
          min_psi=[psi_l,psi_r]
     # *add to container for drawing*
     # *projection is onto the plane spanned by the first two comp.*
	 **if** graphical_output :
          x . append ( v [ 0 ] )
          y . append ( v [ 1 ] )
**print**(”Largest distance:”, max_s)
**print**(”resulting from angle variations:”, np.degrees(max_psi))
**print**(”Smallest distance:”, min_s)
**print**(”resulting from angle variations:”, np.degrees(min_psi))
coi /= **float**(trials)
**print**(”Centroid of estimated object positions: ”, coi)
**print** (”Compared to object at ” , lookat )
**if** graphical_output :
     **import** matplotlib . pyplot as plt
     # *draw eyes and object*
     circle = plt.Circle(eye l , 10.0, fill=False)
     plt.gca().add_patch(circle)
     iris_l = eye_l + 10.0 ∗ ray_l / np.sqrt(ray_l.dot(ray_l))
     circle = plt.Circle(iris_l , 3.0, fill=True, color=’b’)
     plt.gca().add_patch(circle)
     circle = plt.Circle(eye_r , 10.0, fill=False)
     plt.gca().add_patch(circle)
     iris_r = eye_r + 10.0 ∗ ray_r / np.sqrt(ray_r.dot(ray_r))
     circle = plt.Circle(iris_r , 3.0, fill=True, color=’b’)
     plt.gca().add_patch(circle)

     circle = plt.Circle(lookat , 5.0, fill=True, color=’g’)
     plt.gca().add patch(circle)
     plt . scatter (x,y,marker=’. ’ ,color=’r ’ ,alpha=0.02)
     plt.axis(’scaled’)
     plt .xlim([−50,750])
     plt .ylim([−50,50])
     plt . margins (0.2 ,0.2)
     plt.savefig(’vergence.pdf’, bbox_inches=’tight’)
     plt .show()

## Appendix C: Mathematica notebook for analytical analysis

(*Horizontal errors only*)
pl = {−a, 0, 0};
pr = {a, 0, 0};
target = {0, 0, 1};
el = target − pl;
er = target − pr;
epsilonl = {hl , 0, 0};
epsilonr = {hr, 0, 0};
el1= (el + epsilonl)/**Sqrt**[1+(a+hl)ˆ2];
er1=(er + epsilonr)/**Sqrt**[1+(−a+hr)ˆ2];
el2 = (el + epsilonr)/**Sqrt**[1+(a+hr)ˆ2];
er2 = (er + epsilonl)/**Sqrt**[1+(−a+hl)ˆ2];
El1 = **IdentityMatrix**[3]−**Transpose**[{el1}].{el1};
Er1 = **IdentityMatrix**[3]−**Transpose**[{er1}].{er1};
El2 = **IdentityMatrix**[3]−**Transpose**[{el2}].{el2};
Er2 = **IdentityMatrix**[3]−**Transpose**[{er2}].{er2};
p1 = **Inverse**[El1+Er1].(Er1−El1).pr;
p2 = **Inverse**[El2+Er2].(Er2−El2).pr;
**FullSimplify** [0.5*( p1+p2 ) , **Assumptions** −>{}][[−1]]
**Out**[1]= (4. aˆ2)/((2. a+hl−1. hr) (2. a−1. hl+hr))
(*Vertical errors only*)
pl = {−a, 0, 0};
pr = {a, 0, 0};
target = {0, 0, 1};
el = target − pl;
er = target − pr;
epsilonl = {0, vl , 0};
epsilonr = {0, vr, 0};
el1= (el + epsilonl)/**Sqrt**[1+aˆ2+vlˆ2];
er1=(er + epsilonr )/ **Sqrt**[1+aˆ2+vr ˆ2];
el2 = ( el − epsilonr )/ **Sqrt**[1+aˆ2+vr ˆ2];
er2 = (er − epsilonl)/**Sqrt**[1+aˆ2+vlˆ2];
El1 = **IdentityMatrix**[3]−**Transpose**[{el1}].{el1};
Er1 = **IdentityMatrix**[3]−**Transpose**[{er1}].{er1};
El2 = **IdentityMatrix**[3]−**Transpose**[{el2}].{el2};
Er2 = **IdentityMatrix**[3]−**Transpose**[{er2}].{er2};
p1 = **Inverse**[El1+Er1].(Er1−El1).pr;
p2 = **Inverse**[El2+Er2].(Er2−El2).pr;
**FullSimplify** [0.5∗( p1+p2 ) , **Assumptions** −>{}][[−1]]
**Out**[2]= (1. aˆ2 (4. +vlˆ2+2. vl vr+vrˆ2))/(vlˆ2−2. vl vr+vrˆ2+aˆ2 (4. +vlˆ2+2. vl vr+vrˆ2))
